# [18F]FDG-PET/CT-based evaluation of tumor response kinetics during induction chemotherapy and concurrent radiochemotherapy in stage II - III small-cell lung cancer

**DOI:** 10.1007/s00259-025-07658-5

**Published:** 2025-11-17

**Authors:** Christian Hoffmann, Hubertus Hautzel, Marcel Wiesweg, David Kersting, Nika Guberina, Christoph Pöttgen, Martin Metzenmacher, Martin Schuler, Thomas Gauler, Benedikt Höing, Cornelius Kürten, Fabian Doerr, Servet Bölükbas, Natalie Baldes, Marcel Opitz, Aleksandar Milosevic, Felix Nensa, Lale Umutlu, Faustina Funke, Wolfgang P. Fendler, Ken Herrmann, Martin Stuschke, Maja Guberina

**Affiliations:** 1 Department of Radiotherapy, West German Cancer Center, University Hospital Essen, University Duisburg – Essen, Hufelandstrasse 55, Essen, 45147 Germany; 2 NCT West, National Center for Tumor Diseases (NCT), Essen, Germany; 3 German Cancer Consortium (DKTK), Partner Site Essen/Duesseldorf, Essen, Germany; 4 Department of Nuclear Medicine, University Hospital Essen, University Duisburg – Essen, Essen, Germany; 5 Department of Medical Oncology, West German Cancer Center University Hospital Essen, University Duisburg – Essen, Essen, Germany; 6 Department of Otorhinolaryngology, Head and Neck Surgery, West German Cancer Center, University Hospital Essen, University Duisburg – Essen, Essen, Germany; 7 Department of Thoracic Surgery and Thoracic Endoscopy, West German Cancer Center, University Medicine Essen-Ruhrlandklinik, University Duisburg – Essen, Essen, Germany; 8 Department of Diagnostic and Interventional Radiology and Neuroradiolog, University Hospital Essen, University Duisburg – Essen, Essen, Germany; 9 Institute for Artificial Intelligence in Medicine, University Hospital Essen, NCT West, Essen, Germany; 10 Department of Pulmonary Medicine, Section of Interventional Pneumology, West German Cancer Center, University Medicine Essen-Ruhrlandklinik, University Duisburg – Essen, Essen, Germany

**Keywords:** Small cell lung cancer, [^18^F]FDG-PET/CT, Treatment response, Prognostic, Radiotherapy

## Abstract

**Purpose:**

Evaluation of interim-[^18^F]FDG-PET/CT as a prognostic tool in limited disease small cell lung cancer (SCLC).

**Methods:**

We included 35 patients with limited disease SCLC from a prospective institutional registry in this retrospective study. Patients received induction chemotherapy (3–4 cycles) followed by concurrent radiochemotherapy. Baseline [^18^F]FDG-PET/CT was performed before or shortly after start of induction chemotherapy, interim PET/CT was acquired during late induction or concurrent chemoradiotherapy. Maximum standardized uptake value (SUV_max_), metabolic target volume (MTV), and total lesion glycolysis values (TLG) were determined. An exponential decay model with an asymptotic offset was used to describe treatment response over time. Deviations > 2 standard deviations (SD) above model-predicted means after day 30 were considered poor response. Progression-free survival (PFS) was analyzed.

**Results:**

All patients underwent twice-daily radiotherapy to a base dose of 45 Gy. SUV_max_ showed greater inter-patient variability than MTV. Poor treatment response was observed in 17%, 31%, or 14% at the SUV_max_, MTV and TLG endpoints. Deviations > 2 SD from the model in SUV_max_ and TLG were significantly associated with shorter PFS (*p* = 0.0003, *p* = 0.0014); MTV was not prognostic (*p* = 0.2630). Leave-one-out cross-validation (LOOCV) could confirm the prognostic value of the standardized residual SUV_max_ larger than 2 standard deviations above model estimate as negative PFS predictor (*p =* 0.0152, Fishers exact test).

**Conclusion:**

Our decay model enables the characterization of [^18^F]FDG-PET/CT response parameters from scans acquired at variable time points during induction chemotherapy and at start of concurrent radiochemotherapy. Poor SUV_max_ or TLG response was predictive of PFS. Interim-[^18^F]FDG-PET/CT response may guide individualized treatment adaptation.

## Introduction

The optimal treatment strategy for limited disease small cell lung cancer (SCLC) remains undefined. Median overall survival in the favourable arms of recent trials involving irradiated limited disease SCLC patients ranges from 37.2 to 60.7 months [1–3]. Interim [^18^F]FDG-PET/CT may serve as a valuable prognostic tool by enabling early detection of resistant subclones and guiding treatment adaptation.

Two randomized studies have demonstrated a survival benefit from dose escalation in the setting of accelerated hyperfractionation in limited disease SCLC patients. In the first study, radiotherapy was initiated during the second cycle of chemotherapy, whereas in the second study, it commenced during either the first or second cycle [1, 2]. These trials suggest that there is potential for treatment intensification beyond the standard accelerated, hyperfractionated radiotherapy regimen of 45 Gy, delivered as 2 × 1.5 Gy per day, five days a week [4–6]. At our institution, radiotherapy is typically administered during the fourth cycle of chemotherapy. This timing allows for the assessment of tumor response using [^18^F]FDG-PET/CT imaging during both chemotherapy and chemoradiotherapy. It also provides the opportunity for individualized radiotherapy and target volume adaptation based on tumor shrinkage. Such an individualized and personalized approach aims to minimize treatment burden and side effects, particularly in the esophageal region - a critical consideration given the high rate of comorbidities in this patient population.

Data from a randomized trial suggest that restricting the treatment volume to the post-induction tumor volume - after two cycles of chemotherapy - does not significantly impact survival or local control rates but results in significantly lower rates of grade 3 esophagitis [7]. Accordingly, recent international guidelines recommend that radiotherapy can be safely limited to the post-induction chemotherapy extent of the primary tumor and contiguous adherence tissues [8]. The optimal timing of radiotherapy - either early, concurrent with the first cycle of chemotherapy, or delayed - remains controversial, with multiple prospective studies suggesting no significant differences in survival or local control between the two approaches [9–11]. 

A recent meta-analysis demonstrated that high pre-treatment metabolic tumor volume (MTV) was significantly associated with poorer overall survival (OS) and progression-free survival (PFS), while high pre-treatment maximum standardized uptake values (SUV_max_) showed a modest association with worse OS in SCLC patients [12]. However, in the included studies, the [^18^F]FDG-PET/CT was performed only prior to therapy initiation, rendering it unsuitable for treatment adaptation based on treatment response. To date, few studies have investigated early treatment response assessment using [^18^F]FDG-PET/CT in SCLC patients. Van Loon et al. analyzed fifteen patients with limited disease SCLC with [^18^F]FDG-PET/CT scans acquired both before chemotherapy and during or after the first cycle of chemotherapy for radiotherapy planning. Median time interval between PET scans was 20 days (range: 13–39 days). Using proportional hazards analysis, they showed that reductions in MTV correlated with survival [13]. Christensen et al. evaluated early ^18^F-fluorothymidine-PET- (FLT) and diffusion-weighted-magnetic resonance imaging (MRI) at day 14 after start of chemotherapy in comparison to pretreatment [^18^F]FDG-PET/CT in twelve SCLC patients, eleven with extensive disease. They found that responding lesions had significantly lower FLT-SUV_peak_ than non-responding lesions - suggesting potential predictive value [14]. 

We have shown in patients with stage IIIA and IIIB non-small cell lung cancer undergoing interim [^18^F]FDG-PET/CT after induction chemotherapy prior to concurrent radiochemotherapy that post-induction chemotherapy [^18^F]FDG-PET/CT parameters - such as MTV, SUV_max_, and post-treatment maximum TLG - were significantly associated with OS [15]. 

The aim of the present study was to investigate inter-individual variability in [^18^F]FDG-PET/CT treatment response in stage II-III (limited disease) SCLC patients. Such variability is a prerequisite for the development of a predictive model. In a secondary analysis, we evaluated whether deviations from the model predictions were of prognostic significance. Given the limited follow-up period, PFS was selected as the study endpoint.

## Methods

This is a retrospective observational study based on a prospective institutional registry (Clinical trial registration: 18–8364-BO, 23–11560-BO, 24–11866-BO). The study was conducted in accordance with the Declaration of Helsinki. The study was approved by the ethics committee of the Medical Faculty of the University Duisburg-Essen.

### Patient selection

We included patients with histologically proven and fully staged limited disease SCLC. All patients gave their written informed consent, to be included into the prospective institutional registry approved by the local ethics committee (18–8364-BO). Patients received imaging and treatment according to written institutional standards. This retrospective analysis from the registry was additionally approved by the ethics committee (23–11560-BO). Staging included, as per standard, EBUS-guided histology with systematic mediastinal lymph node sampling, brain MRI, and an [^18^F]FDG-PET/CT scan. Only patients treated with curative intent between 2019 and 2024 were eligible. Patients were identified through our in-house database. Inclusion criteria required that an initial [^18^F]FDG-PET/CT scan for staging had been performed either before or shortly after the start of induction chemotherapy, and a second [^18^F]FDG-PET/CT scan (interim staging) was conducted during the final cycles of induction chemotherapy or during radiochemotherapy. The interim [^18^F]FDG-PET/CT was performed as part of routine clinical care for radiotherapy treatment planning. The [^18^F]FDG-PET/CT interim staging had to be performed before the completion of radiochemotherapy. Further details regarding the temporal sequence of the [^18^F]FDG-PET/CT scans are provided in the Results section, under point 3.2. To ensure comparability, all PET/CT procedures had to be conducted at our hospital. Blood glucose levels were measured prior to the [^18^F]FDG-PET/CT scan and were below 160 mg/dL. Diabetic patients were eligible. To ensure diagnostic quality, international procedural guidelines were followed. Patients with a history of previous malignancies were not included in the study [16, 17]. Patients with a history of previous malignancies were not included in the study. In accordance with international guidelines, patients were required to have received at least four full cycles of chemotherapy. Radiotherapy started routinely with the last chemotherapy cycle. The standard chemotherapy protocol prescribed cisplatin (25 mg/m² body surface area (BSA), days 1–4) and etoposide (100 mg/m² BSA, days 2–4) during induction chemotherapy. Concurrent with radiotherapy, cisplatin (40–50 mg/m² BSA, days 1 and 8) and etoposide (80–100 mg/m² BSA, days 3–5) were administered. Doses were occasionally adjusted based on patient fitness. In cases of contraindications, cisplatin was replaced with carboplatin. All patients received radiotherapy using volumetric arc therapy to a standard dose of 45 Gy, administered in 1.5 Gy fractions twice daily. In addition to the standard dose, an individualized boost up to 60 Gy was offered to patients with residual tumor on interim [^18^F]FDG-PET/CT or those deemed to be at higher risk for local recurrence, following a shared decision-making process with the patient and in accordance with the National Interdisciplinary Guidelines, using either a hyperfractionated or normofractionated approach [18]. The median total dose was 55 Gy. Prophylactic cranial irradiation was routinely offered. Following publication of the ADRIATIC study, durvalumab consolidation has been offered if reimbursed by the patient’s health insurance [3]. 

### Imaging

Radiological and functional imaging data were acquired using the ARIA radiotherapy planning system (Varian Medical Systems, Palo Alto, CA, Version 16.00). All [^18^F]FDG-PET/CT procedures complied with the international guidelines proposed by the European Association of Nuclear Medicine [16, 17]. The methodological details have been previously described [15]. Briefly, the MTV was delineated on the [^18^F]FDG-PET/CT dataset via the “PET subvolume thresholding” module set to a uniform threshold, reviewed individually andadapted if appropriate [19]. Automated segmentations were subsequently refined through manual editing on co-registered CT images to exclude adjacent non-malignant mediastinal structures. In patients with multiple lesions, such as a primary tumor and lymph node metastases, the previously mentioned threshold approach was applied to each lesion individually. Subsequently, a compound structure representing the sum of all lesions was constructed and further analyzed. Quantification of SUV_max_ was based on body weight–normalized standardized uptake values, derived from the earliest time point of image acquisition. Total lesion glycolysis was computed as the product of MTV and SUV_max_. Given the high collinearity between SUV_max_ and SUV_mean_, SUV_mean_ was not independently assessed [20, 21]. 

### Statistics

To describe the signal decay of SUV_max_, MTV, and TLG over time during therapy, we employed an exponential decay model (model 1) with an asymptotic offset. The model used (exemplified here for SUV) was:

SUV(time) = Bi × [(1 – c) × exp(–a × time) + c] model (1).

In this model, *–a* represents the common decay parameter across all patients, *c* is the common asymptotic offset, and *Bi* is a patient-specific scaling factor. The *Bi* parameters as classification parameters were optimized conditional on the respective patient. All numerical analyses were performed using the SAS statistical software system (SAS/STAT 15.1, SAS institute Inc. Cary, NC; USA). The model was fitted to the raw data using non-linear least squares approximation (Procedure NLIN, SAS). Time was measured from the start of induction chemotherapy (defined as day 0). Since the initial [^18^F]FDG-PET/CT scans were typically not performed precisely on day 0 due to the nature of data acquisition during clinical routine (interquartile range: day − 19 to day − 1), we assumed that the SUV_max_ remained constant within this range. For the MTV, we assumed that the tumor’s growth rate before therapy is matched by the rate of shrinkage after the start of chemotherapy. Based on this assumption, we adjusted the time point of the initial PET/CT from day “–x” to day “+” down to a date as early as day − 21 before the start of chemotherapy. We assigned day 21 to the 17% of patients with even earlier scans. This backdating method was also applied to the TLG, which was defined as the product of SUV_max_ and MTV. Accordingly, TLG was treated as proportional to both variables.

To investigate the prognostic value of interpatient variability in PET-derived parameters, we developed model (1) using SUV_max_, MTV, and TLG data from all included patients. As a prognostic factor, any deviation of a measured PET parameter from the model’s mean predicted value by more than two standard deviations (SD) above the mean, occurring after day 30 from the start of induction chemotherapy, was considered a negative prognostic indicator. For parameters exhibiting sufficient interindividual variability - defined as more than 15% of patients showing a positive prognostic indicator - and showing an association with prognosis, we performed a leave-one-out cross-validation (LOOCV) model (1). Due to limited follow-up duration, PFS was selected as an early endpoint. A PFS event was defined as any tumor relapse, whether local, locoregional, or distant. For the LOOCV procedure, each patient was excluded in a loop from the training dataset. The model was then built using the remaining data. Parameters „a“ and „c“ were taken from this model, and parameter „Bi“ was fitted to the two or more available data points of the left-out patient. This process was repeated for all patients. A negative prognostic factor was assigned if a measured PET parameter for the left-out patient exceeded the model prediction by more than two standard deviations. To display differences in PFS, the Kaplan–Meier method was used. The level of significance was determined using the log-rank test. For the LOOCV prediction model, Fisher’s exact test was applied to assess the significance of the association between a leave-one-out observation that exceeded the model-predicted value by more than two standard deviations and the occurrence of a PFS event. Logistic regression and receiver operator curve analysis was performed with the SAS Procedure logistic. Median follow-up times were calculated according to the reverse Kaplan-Meier Method [22]. 

## Results

### Patients

Altogether, 35 patients treated between 2019 and 2024 met the study’s inclusion criteria. Table 1 summarizes the patient characteristics. In sum 32 of the 35 patients received three cycles of induction chemotherapy and a total of four chemotherapy courses. Three patients received four cycles of induction chemotherapy and a total of five chemotherapy courses. All patients received radiotherapy using volumetric arc therapy to a standard dose of 45 Gy, administered in 1.5 Gy fractions twice daily and 23 patients received a radiotherapy boost up to a median total dose of 55 Gy. Prophylactic cranial irradiation was routinely offered. Seven patients declined this treatment. Nine patients received durvalumab consolidation.

**Table 1:** Patients characteristics, ECOG = Eastern cooperative oncology Group, RT = radiotherapy, Gy = Gray

Characteristic	No. of patients/Value
Age at diagnosis (years)	
Median	64
Range	44–84
Sex	
Female	19
Male	16
Follow-up (months)	
Median	5
Range	1–41
ECOG	
0	4
1	24
2	7
Stage	
IIA	1
IIB	3
IIIA	7
IIIB	15
IIIC	9
Cylces of induction chemotherapy	
3	32
4	3
Cylces of chemotherapy (total)	
4	32
5	3
Concurrent radiochemotherapy	
Yes	35
No	0
Hyperfractionated accelerated radiotherapy to 45 Gy, twice daily	
Yes	35
No	0
Total RT dose (Gy)	
Median	55
5th-95th percentile range	45–60
Prophylactic cranial irradiation	
Yes	28
No	7
Durvalumab consolidation	
Yes	9
No	26

Median time of follow-up was 7.8 months (95% CI: 5.6–9.6 months). Patients fasted for more than 6 h prior to the injection of (^18^F)-fluoro-2-deoxyglucose. Blood glucose levels were below 160 mg/dL at the time of administration. Diagnostic PET/CT images were acquired 65 ± 11 min after the injection of 290 ± 46 MBq FDG, using one of the following systems: Biograph mCT 128 (Siemens Healthineers, Erlangen, Germany), Vision 600 (Siemens Healthineers), or Vereos (Philips Healthcare).

### Decay modeling of SUV_max_, MTV and TLG

At initial [^18^F]FDG-PET/CT staging, patients presented with a median lesion SUV_max_ of 15.2 (range: 5.7–28.0). Relative to the start of chemotherapy, the initial [^18^F]FDG-PET/CT scan was conducted at a median of day − 7 (10th −90th percentile: day − 33 to day + 5). In total, six patients received the staging PET scan after the initiation of chemotherapy, but none of these patients had received more than one cycle of chemotherapy at that time. Seven patients underwent a supplementary [^18^F]FDG-PET/CT as an early interim scan during the course of chemotherapy for reassessment. The interim PET/CT for radiotherapy planning was performed at a median of day 86 (10th −90th percentile: day 69 to day 115). This interim [^18^F]FDG-PET/CT was performed either before (*n* = 13) or shortly after the start of radiochemotherapy (*n* = 22).

**Fig. 1:**
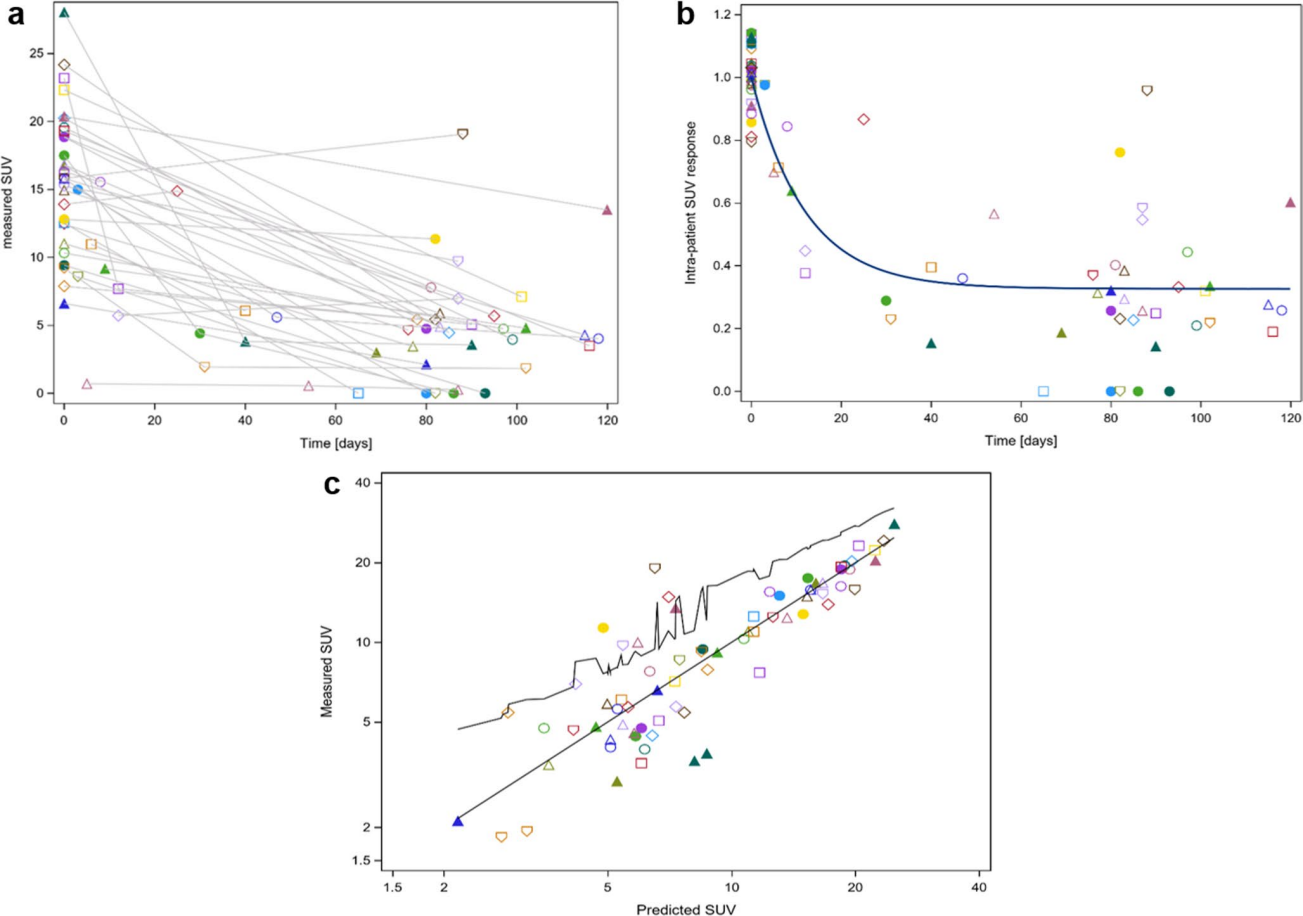
**a** Raw measured SUV_max_ over time (days) after the initiation of induction chemotherapy. Different symbols represent data from individual patients. **b**: Mono-exponential decay model (1) with a common offset, modeling SUV_max_ decay as a function of time after the start of induction chemotherapy. Both measured and predicted values were normalized using the patient-specific scaling parameter Bi. **c**: Measured versus predicted SUV_max_ based on model (1). The upper serrated line connects the upper bound of the 95% confidence interval for the mean predicted values. SUV_max_ = maximum standardized uptake value, Bq/cm^3^ = Becquerel per cubic centimeter

Figure 1a shows the raw SUV_max_ measurements over time following the initiation of induction chemotherapy. These values were used to construct the mono-exponential decay model (1) (Fig. 1b). A patient-specific scaling parameter, *Bi*, was applied for normalization. Notably, considerable inter-subject variability was observed in the SUV_max_ response, a necessary condition for developing a predictive model for individualized therapy. Figure 1c displays the measured versus predicted SUV_max_ over time, as derived from model 1, demonstrating good agreement between observed and predicted data.

**Fig. 2:**
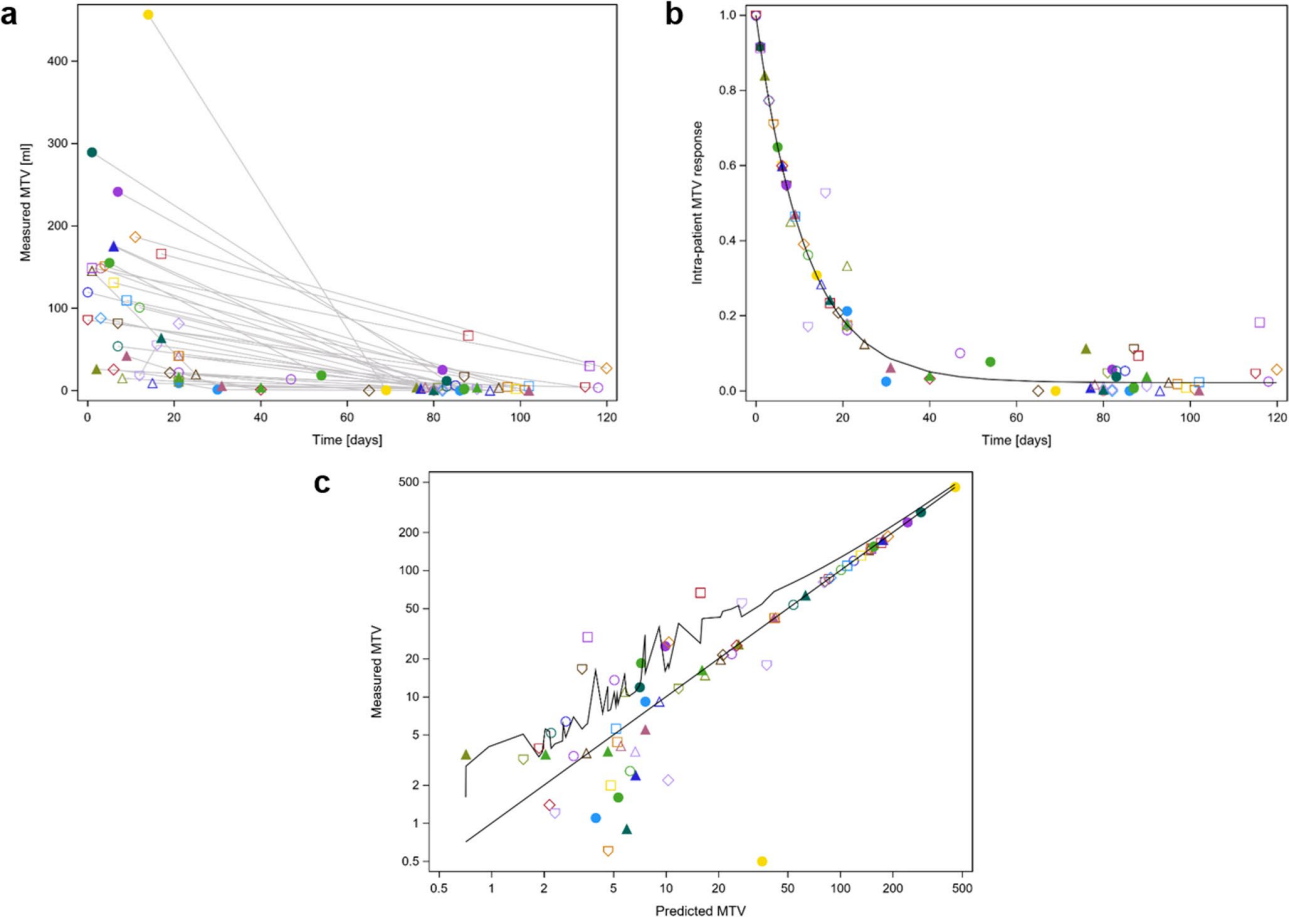
**a** Raw measured MTV values (ml) over time (days) after the initiation of induction chemotherapy. Different symbols represent data from individual patients. **b**: Monoexponential decay model (1) with a common offset, modeling MTV decay as a function of time after the start of induction chemotherapy. Both measured and predicted values were normalized using the patient-specific scaling parameter Bi. Of note, the MTV signal decays more uniformly than maximum standardized uptake values. **c**: Measured versus predicted MTV (ml) values based on model (1). The upper serrated line represents the upper bound of the 95% confidence interval for the mean predicted values. MTV = metabolic tumor volume, ml = milliliter

**Fig. 3:**
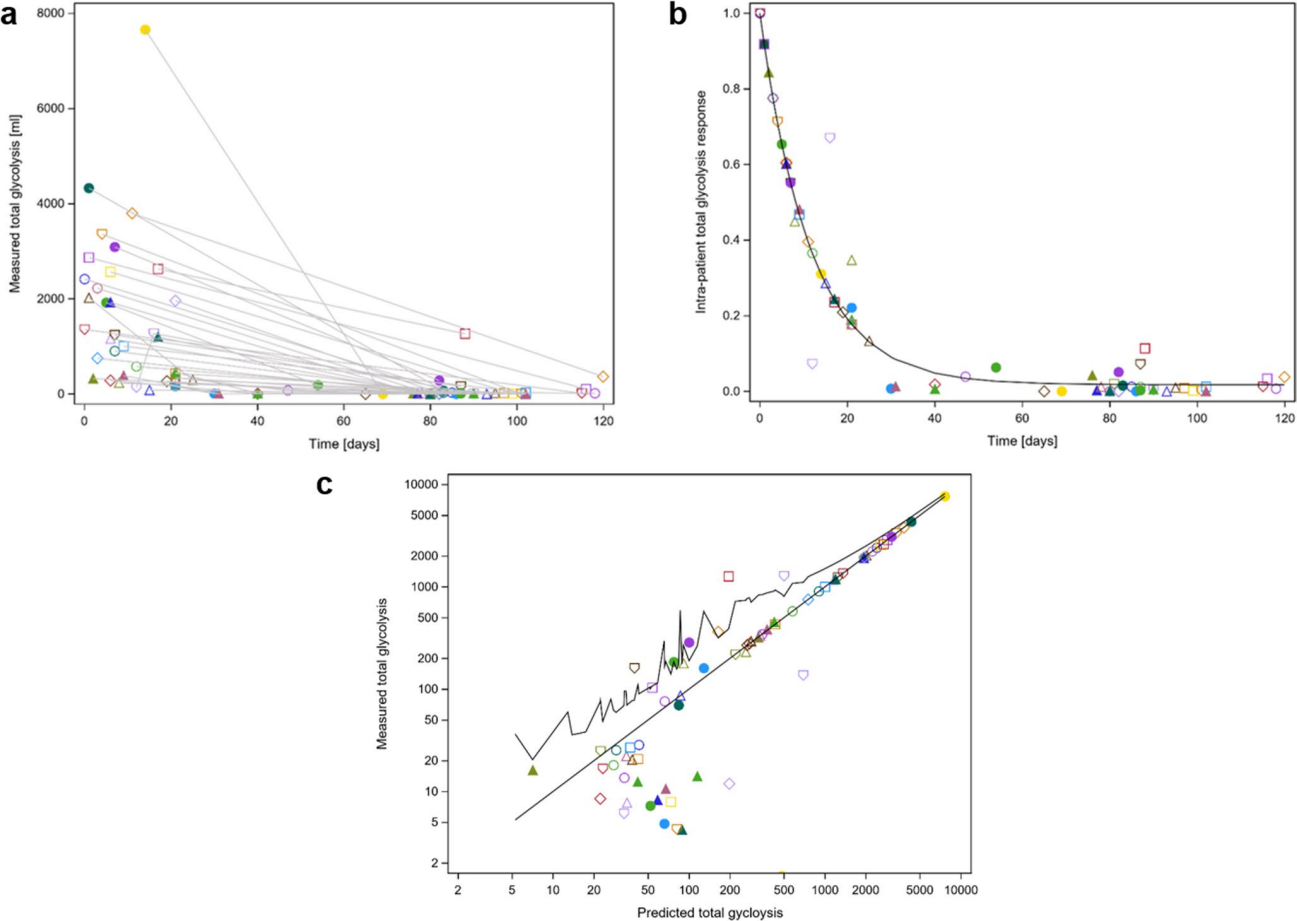
**a** Raw measured TLG values (ml) over time (days) after the initiation of induction chemotherapy. Different symbols represent data from individual patients. **b**: Monoexponential decay model (1) with a common offset, modeling TLG decay as a function of time after the start of induction chemotherapy. Both measured and predicted values were normalized using the patient-specific scaling parameter Bi. **c**: Measured versus predicted TLG (ml) values based on model (1). The upper serrated line represents the upper bound of the 95% confidence interval for the mean predicted values. TLG = total glycolisis, ml = milliliter

The same model was applied to analyze the MTV decay. Patients presented at the staging with a median initial MTV of 85.7 ml (range: 9.2–456.6 ml). In contrast to the SUV_max_, the MTV showed a more uniform decay pattern (Fig. 2a-c). Figure 3 **a-c** presents the corresponding analysis for the TLG. Patients exhibited a median TLG at the staging PET/CT of 1199.2 ml (range: 86.7–7657.6 ml). As for the SUV_max_ there was good agreement between observed and predicted data for MTV and TLG.

Table 2 summarizes half time of decrease values for SUV_max_, MTV and TLG and reports the value of the asymptotic relative residual offset, represented by *c* in the equation described in the statistical analysis section.

**Table 2:** Model parameters were obtained from the Raw measured SUV_max_, MTV and TLG data, as shown in Figs. 1a and 2a, and 3a. The half-time of decrease is given by *T1/2 = -ln2/a*, based on model (1) described in the methods section. The asymptotic offset corresponds to the parameter *c* from model (1). The R-squared value represents the coefficient of determination, indicating the proportion of variance explained by the model. SUV_max_ = maximum standardized uptake value, MTV = metabolic tumor volume, TLG = total lesion Glycolysis

PET-parameter	Half time of decrease (days, 95% CI)	Asymptotic offset (95% CI)	*R*-squared
SUV_max_	8.28 (5.09–22.2)	0.326 (0.276–0.376)	0.9431
MTV	7.86 (5.87–11.9)	0.022 (0.013–0.031)	0.9901
TLG	8.00 (5.49–14.8)	0.017 (0.007–0.027)	0.9856

### Prognostic value of interindividual treatment response deviation

**Fig. 4:**
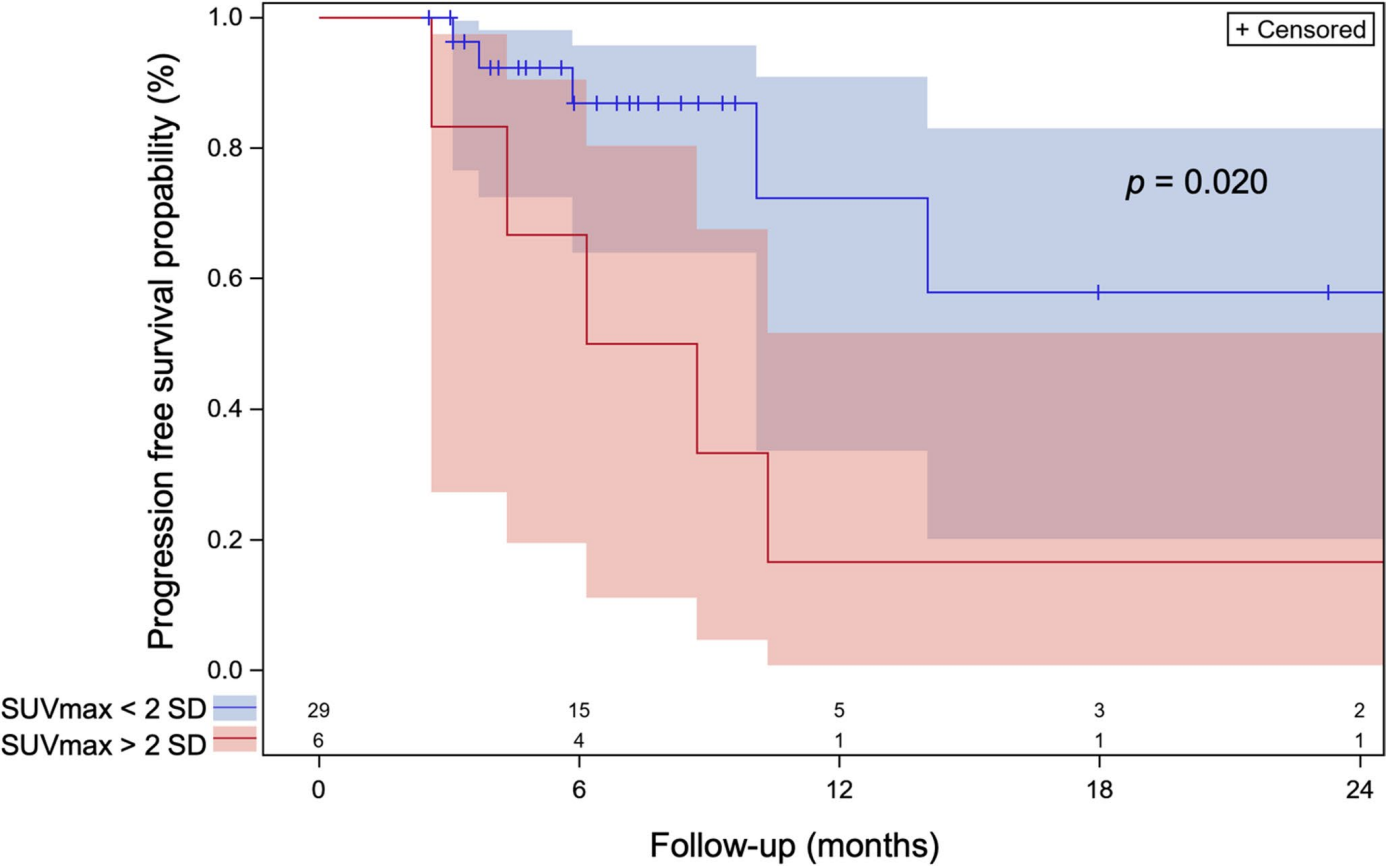
Kaplan–Meier PFS curves for patients with SUV_max_ greater than two standard deviations above the mean predicted values at times > 30 days from treatment initiation (red step function) versus those who did not meet this criterion (blue step function). The PFS curves differ significantly (*p* = **0.020**, log-rank test). Numbers above the x-axis indicate the number of patients at risk at each time point. PFS = progression-free survival, SUV_max_ = maximum standardized uptake value, SD = standard deviation

The aforementioned data were used to predict treatment response to induction chemotherapy. For each patient, treatment responses for SUV_max_, MTV, and TLG derived from the radiotherapy planning [^18^F]FDG-PET/CT scan were classified as either above or within/below 2 SD of the model’s mean estimate. Patients were required to have at least two observations from different time points for inclusion in this analysis. A value above this threshold was considered a risk factor for relapse. The proportion of patients with a positive risk indication was 17%, 31%, and 14% for the SUV_max_, MTV, and TLG endpoints, respectively. These results were cross-tabulated against the PFS status (Table 3). A significantly increased risk for a PFS event was associated with a small treatment response above 2 SD of the model’s mean of measured SUV_max_ and TLG values (*p =* 0.0003 and *p =* 0.0014, Fisher’s exact test). In contrast, a small response of MTV did not show a significant association with PFS outcome (*p =* 0.2630, Fisher’s exact test). Kaplan–Meier analysis (Fig. 4) demonstrated significantly different PFS propability between patients with SUV_max_ response values more than 2 SD above the predicted mean and those within/below this threshold (*p =* 0.020, log-rank test). Figure 4 PFS for all patients at 12 months follow-up was 0.54 (95% CI: 0.26–0.75), the median PFS time was 14.0 months (95% CI: 8.7–42.7 months). The area under the curve for predicting a PFS event using the standardized SUV_max_ residual, with a risk cut-off defined as 2 SD above the predicted values, was 0.77 (95% CI: 0.62–0.93). All patients with SUV_max_ exceeding 2 SD above the model prediction received a radiotherapy boost beyond 45 Gy, while 80% of the remaining patients who relapsed also received a boost.

**Table 3:** SUV_maxpd_, MTV_pd_ and TLG_pd_ using predating for data from the initial PET/CT. For the different parameters, the number of patients with measured values at day > 30 day from start of chemotherapy above or below the model prediction + 2 SD of the mean as a risk factor was given and cross tabulated with the patient having a PFS event or not. The association between PFS event and a positive risk factor from the PET/CT end point was analysed with the fisher’s exact test. SUV_maxpd_ = pre-dated maximum standardized uptake value, MTV_pd_ = pre-dated metabolic tumor volume, TLG_pd_ = pre-dated total lesion glycolysis, SD = standard deviation, PFS = progression free survival

Parameter	PFS-event	Inliers below model estimate + 2 SD	Outliers above model estimate + 2 SD	*p*-value Fisher’s exact test
SUV_maxpd_	no	24	0	**0.0003**
	yes	5	6	
MTV_pd_	no	18	6	0.2630
	yes	6	5	
TLG_pd_	no	24	0	**0.0014**
	yes	6	5	

For comparison with other studies, we also determined the association of the logarithms of the pre-treatment MTV or TLG, and the pre-treatment SUV_max_ with PFS, using the proportional hazards model. All pre-treatment PET parameters were extrapolated to time = 0 using the presented decay model. The respective hazard ratios were 1.137 (95% CI: 0.575–2.247), 0.964 (95% CI: 0.527–1.761), and 0.906 (96 CI: 0.784–1.047), respectively. Therefore, none of the pre-treatment PET parameters was prognostic for PFS. In total, nine patients experienced a disease recurrence. Among them, two had an in-field relapse only, two had a combination of in-field/locoregional and distant relapse, and five presented with a distant relapse only. Adjuvant immunotherapy was not prognostic for PFS in this study (*p* = 0.70, log-rank test), nor was it associated with poor SUV_max_ response to induction chemotherapy (*p* = 0.21, Fisher’s exact test). Adjuvant immunotherapy was introduced for all patients following the availability of the ADRIATIC data in 2024 [3]. In addition, the application of prophylactic cranial irradiation was not a prognostic factor for PFS (*p* = 0.98, log-rank test).

**Fig. 5:**
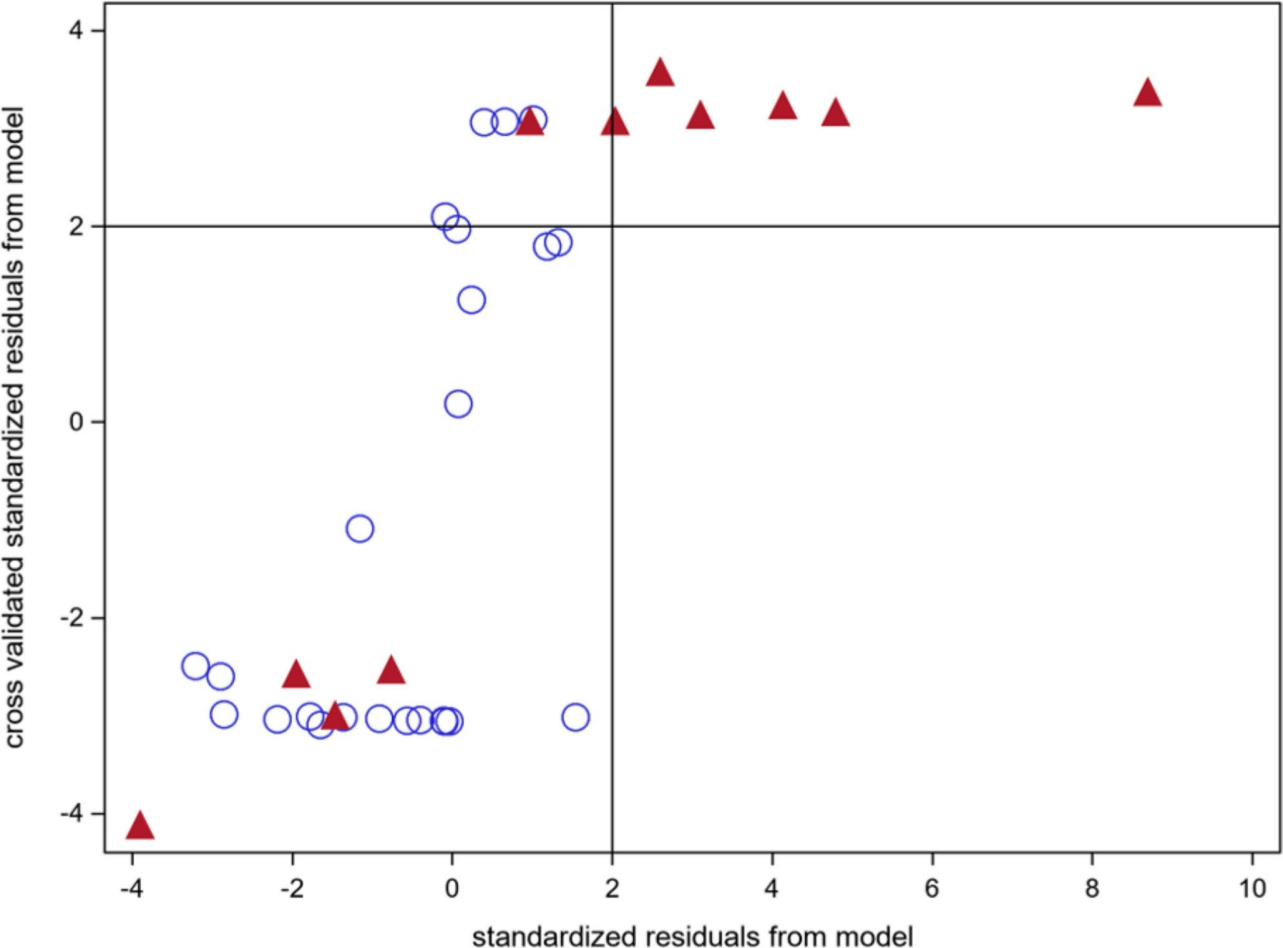
Leave-one-out cross-validated SUV_max_ predictions from model (1) compared to SUV_max_ predictions based on the full dataset, shown as standardized residuals ((measured minus predicted values) divided standard deviation of the predicted value). There is a strong rank correlation (rs = 0.72, 95% CI: 0.51–0.85). Filled upward-pointing triangles represent observations from patients who experienced a PFS event, open circles represent patients without an event during follow-up. There was an association between a leave one out observation more than 2 standard deviations above the predicted value from the cross validated model and the occurrence of a PFS event (*p =* 0.0152, Fisher’s exact test). At least two observations from different time points per patient were required. SUV_max_ = maximum standardized uptake value; PFS = progression-free survival

LOOCV SUV_max_ predictions from model (1) were compared with SUV_max_ predictions derived from the full dataset, expressed as standardized residuals (Fig. 5). A strong rank correlation was observed between the standardized residuals using cross validation or the full data set for model approximation (rs *p* = 0.72, 95% CI: 0.51–0.85). Observations associated with a PFS event also showed more likely standardized residuals greater than two standard deviations above the predicted value from the cross-validated model. This association was statistically significant (*p =* 0.0152, Fisher’s exact test).

## Discussion

The prognosis of patients with limited disease SCLC remains poor. Median progression-free survival rates in the favourable arms of recent trials of irradiated limited disease SCLC patients range from 37.2 to 60.7 months [1–3]. Across several randomized trials in limited disease SCLC, distant progression - particularly cerebral metastases - remains an important mode of failure [1, 2, 7, 23]. The median metastatic-free survival ranged from 16.6 months to 40.2 months in various studies, unaffected by radiation dose [1, 2, 4, 23]. The median PFS time in the present study was 14.0 months (95%-CI: 8.7–42.7 months) which falls within the range of outcomes reported in the large, randomized trials using accelerated, hyperfractionated radiotherapy and concurrent chemotherapy, especially when considering the respective confidence intervals. In the ADRIATIC trial, median progression free survival was 16.6 months (95 CI: 10.2–29.2 months) in the durvalumab arm and 9.2 months (95% CI: 7.4–12.9 months) in the placebo arm [3]. Median PFS was 15.4 months (95% CI: 13.7–19.8 months) in the twice-daily arm of the CONVERT trial. The median PFS in the standard 45 Gy total dose arms of the two radiation dose escalation trials from Gronberg et al. and Yu et al. were 10.9 months (95% CI: 8.7–13.1 months) and 16.7 months (95% CI: 10.7–22.7 months), respectively. Median PFS in the dose escalated arms to 60 Gy–54 Gy were 18.6 months (95% CI: 7.3–30.0 months) and 30.5 months (95% CI: 14.5–46.5 months) in the aforementioned trials [1, 2, 4]. 

In the present study, nine patients experienced a disease recurrence. Among them, two (5.7%) had an in-field relapse only, two had a combination of in-field/locoregional and distant relapse, and five presented with a distant relapse only. However, the follow-up is short. All poor responders in the interim [^18^F]FDG-PET/CT and both patients with an isolated in-field recurrence received a radiotherapy boost. Local control is comparable to data from other studies. Turrisi reported a 30% crude rate of isolated local recurrences in the twice-daily irradiation arm [5]. Hu reported crude isolated infield recurrence rates of 7.8% in the arm with reduced post-chemotherapy target volume and 14.9% in the control arm using a pre-chemotherapy target volume [7]. Gronberg reported a crude incidence of 35% in-field recurrences in the 45 Gy arm and of 21% in the 60 Gy arm [2]. 

Radiotherapy dose-escalation has been shown to increase PFS in two randomised trials [1, 2]. Immunotherapy consolidation has shown this effect in one randomized trial [3]. Both therapy intensification strategies demonstrate considerable potential and warrant further optimization in subsequent clinical trials. A key avenue for future therapeutic development is the individualization of treatment based on a predictive test, thereby minimizing toxicity in patients for whom intensification is unlikely to confer additional benefit.

The rates of esophagitis and pneumonitis of grade 3–4 were not increased in the high dose arms of the radiation dose escalation trials. Gronberg et al. reported grade 3–4 esophagitis rates of 21% versus 18% in the 60 Gy and 45 Gy total dose groups, respectively (*p =* 0.83), and pneumonitis grade 3–5 rates of 4% vs. 0% [2, 23]. Similarly, Yu et al. reported grade 3–4 esophagitis in 13% of patients in the 54 Gy group compared to 12% in the 45 Gy group (*p =* 0.84), and pneumonitis rates of 5% versus 6%, respectively (*p =* 0.66) [1]. Initiating thoracic radiotherapy with the third or fourth cycle of induction chemotherapy, rather than the first cycle, allows for reduced target volumes that are adapted to the regressed primary tumor. Hu et al. showed in a prospective randomized trial that restricting the target volume to the post-chemotherapy tumor volume after two cycles of induction chemotherapy significantly reduces the rate of grade 3 esophagitis but has no detrimental effects on local PFS [7]. The total rate of pneumonitis was 38.2% in the durvalumab group versus 30.2% in the placebo group of the ADRIATIC trial [3]. Because durvalumab may influence the risk of radiation-induced pneumonitis, careful selection of the target volume is required [24]. 

Initiating radiotherapy concurrently with the third or fourth course of cisplatin/etoposide chemotherapy allows for the assessment of the predictive value of the PET response during induction chemotherapy alone. The results of the present study show that a poor SUV_max_ or TLG response on post-induction [^18^F]FDG-PET/CT is of predictive value. Data on the predictive value of repeated PET parameters during therapy are limited. Kim et al. investigated a cohort of both extensive and limited disease SCLC patients and found by multivariate analyses, that the percentage change between pre- and post-treatment SUV_peak_ was an independent prognostic factor for OS, while post-treatment MTV was also predictive for PFS [25]. Van Loon et al. selected fifteen patients with stage I-III SCLC treated with concurrent chemoradiotherapy who had [^18^F]FDG-PET/CT scans available both before the start of chemotherapy and after or during the first chemotherapy cycle, but prior to radiotherapy. MTV was calculated for the primary tumor and involved nodal stations using both 40% and 50% of SUV_max_ thresholds. Changes in MTV40 and MTV50 were significantly associated with patient survival [13]. Christensen et al. evaluated early treatment response in 12 SCLC patients using ^18^F-fluorothymidine-(FLT)-PET/at a median interval of 4.5 days (range: 1–9 days) after start of chemotherapy and compared the MTV, SUV_max_ and TLG values for FLT-PET with those values from pretreatment FDG-PET. FLT-SUV_max_ measured early after the start of chemotherapy were significantly lower in lesions that ultimately responded compared to non-responding lesions. This finding indicates that early post-treatment proliferation, as assessed by FLT-PET, may serve as a predictor of treatment response [14]. 

Most studies on the predictive value of [^18^F]FDG-PET/CT have focused on pre-treatment [^18^F]FDG-PET/CT parameters. Choi et al. reported on 50 patients with limited disease SCLC, where the pre-treatment SUV_max_ of the primary tumor was an independent predictor of OS. MTV and TLG were significant prognostic factors in univariate analysis, but not in multivariate analysis [26]. Chang et al. studied 30 patients with limited disease SCLC and found that a pre-treatment SUV_max_ above a certain cut-off value was a positive predictor of PFS in a multivariate analysis, while an elevated MTV was predictive of OS. TLG was found to be predictive of OS in univariate analysis [27]. In a larger study of 129 patients with limited disease SCLC, Fu et al. identified the MTV prior to treatment initiation as an independent factor for both PFS and OS [28]. A recent meta-analysis showed that high pre-treatment MTV was associated with significantly worse OS and PFS (pooled HR for OS: 2.83 [95% CI: 2.00–4.01.00.01], *p* < 0.0001; pooled HR for PFS: 3.22 [95% CI: 1.96–5.28], *p* < 0.0001), although high heterogeneity among the included studies was found [12]. The same meta-analysis revealed a modestly increased hazard ratio for OS in patients with high pre-treatment SUV_max_ (pooled HR: 1.50 [1.17–1.91], *p =* 0.001), but pre-treatment SUV_max_ was not prognostic for PFS (pooled HR: 1.24 [0.94–1.63], *p =* 0.13) [12]. In contrast to these studies, the present study found a higher predictive value of the SUV_max_ or TLG PET decline during therapy than that of the respective pretreatment parameters. Our study group published similar results on the predictive value of the SUV_max_ and TLG response on pre-treatment and post-induction chemotherapy [^18^F]FDG-PET/CT, following the treatment sequence of induction chemotherapy and definitive radiochemotherapy or neoadjuvant radiochemotherapy and resection in patients with locally advanced non-small cell lung cancer [15, 29–33]. As described by Berghmans et al., the treatment of lung cancer patients remains highly challenging and requires close multidisciplinary cooperation [34]. Only through strengthened collaboration among the involved specialties can new, pioneering insights for this challenging patient population be achieved.

The small cohort size (*n* = 35) and limited follow-up (median: 7.8 months) limits the generalizability of our findings. PFS was selected as the primary endpoint due to the short follow-up times. Moreover, the generalizability of these findings is constrained by the retrospective design of the study and the heterogeneity of the patient cohort with regard to radiation dose and the administration of durvalumab consolidation. This exploratory study found an association between SUV_max_ and TLG response observed on interim [^18^F]FDG-PET/CT and PFS, but analysis in a larger independent cohort with extended follow-up is essential to validate these findings and assess the impact on OS.

## Conclusion

The decay model developed in this study allows for the characterization of [^18^F]FDG-PET/CT response parameters from scans obtained at varying time points during induction chemotherapy and at start of concurrent radiochemotherapy in limited disease small-cell lung cancer patients. Notably, poor SUV_max_ or TLG response was predictive of progression-free survival. These exploratory findings point out that [^18^F]FDG-PET/CT response assessment could serve as a valuable tool to guide risk stratification and treatment adaptation in future clinical trials, ultimately aiming to improve personalized therapeutic strategies.

## Data Availability

The datasets generated and analyzed during the current study are not publicly available due to the fact that they contain personal clinical data. They are available from the corresponding author on reasonable request in an anonymized form.

## References

[1] Yu J, Jiang L, Zhao L, Yang X, Wang X, Yang D, et al. High-dose hyperfractionated simultaneous integrated boost radiotherapy versus standard-dose radiotherapy for limited-stage small-cell lung cancer in China: a multicentre, open-label, randomised, phase 3 trial. Lancet Respir Med. 2024;12:799–809. 10.1016/S2213-2600(24)00189-9. 39146944 10.1016/S2213-2600(24)00189-9

[2] Gronberg BH, Killingberg KT, Flotten O, Brustugun OT, Hornslien K, Madebo T, et al. High-dose versus standard-dose twice-daily thoracic radiotherapy for patients with limited stage small-cell lung cancer: an open-label, randomised, phase 2 trial. Lancet Oncol. 2021;22:321–31. 10.1016/S1470-2045(20)30742-7. 33662285 10.1016/S1470-2045(20)30742-7

[3] Cheng Y, Spigel DR, Cho BC, Laktionov KK, Fang J, Chen Y, et al. Durvalumab after chemoradiotherapy in limited-stage small-cell lung cancer. N Engl J Med. 2024;391:1313–27. 10.1056/NEJMoa2404873. 39268857 10.1056/NEJMoa2404873

[4] Faivre-Finn C, Snee M, Ashcroft L, Appel W, Barlesi F, Bhatnagar A, et al. Concurrent once-daily versus twice-daily chemoradiotherapy in patients with limited-stage small-cell lung cancer (CONVERT): an open-label, phase 3, randomised, superiority trial. Lancet Oncol. 2017;18:1116–25. 10.1016/S1470-2045(17)30318-2. 28642008 10.1016/S1470-2045(17)30318-2 PMC5555437

[5] Turrisi AT 3rd, Kim K, Blum R, Sause WT, Livingston RB, Komaki R, et al. Twice-daily compared with once-daily thoracic radiotherapy in limited small-cell lung cancer treated concurrently with cisplatin and etoposide. N Engl J Med. 1999;340:265–71. 10.1056/NEJM199901283400403. 9920950 10.1056/NEJM199901283400403

[6] Bogart J, Wang X, Masters G, Gao J, Komaki R, Gaspar LE, et al. High-dose once-daily thoracic radiotherapy in limited-stage small-cell lung cancer: CALGB 30610 (Alliance)/RTOG 0538. J Clin Oncol. 2023;41:2394–402. 10.1200/JCO.22.01359. 36623230 10.1200/JCO.22.01359 PMC10150922

[7] Hu X, Bao Y, Xu YJ, Zhu HN, Liu JS, Zhang L, et al. Final report of a prospective randomized study on thoracic radiotherapy target volume for limited-stage small cell lung cancer with radiation dosimetric analyses. Cancer. 2020;126:840–9. 10.1002/cncr.32586. 31714592 10.1002/cncr.32586

[8] Le Pechoux C, Faivre-Finn C, Ramella S, McDonald F, Manapov F, Putora PM, et al. ESTRO ACROP guidelines for target volume definition in the thoracic radiation treatment of small cell lung cancer. Radiother Oncol. 2020;152:89–95. 10.1016/j.radonc.2020.07.012. 32673777 10.1016/j.radonc.2020.07.012

[9] Work E, Nielsen OS, Bentzen SM, Fode K, Palshof T. Randomized study of initial versus late chest irradiation combined with chemotherapy in limited-stage small-cell lung cancer. Aarhus Lung Cancer Group. J Clin Oncol. 1997;15:3030–7. 10.1200/JCO.1997.15.9.3030. 9294465 10.1200/JCO.1997.15.9.3030

[10] Skarlos DV, Samantas E, Briassoulis E, Panoussaki E, Pavlidis N, Kalofonos HP, et al. Randomized comparison of early versus late hyperfractionated thoracic irradiation concurrently with chemotherapy in limited disease small-cell lung cancer: a randomized phase II study of the Hellenic cooperative oncology group (HeCOG). Ann Oncol. 2001;12:1231–8. 10.1023/a:1012295131640. 11697833 10.1023/a:1012295131640

[11] Sun JM, Ahn YC, Choi EK, Ahn MJ, Ahn JS, Lee SH, et al. Phase III trial of concurrent thoracic radiotherapy with either first- or third-cycle chemotherapy for limited-disease small-cell lung cancer. Ann Oncol. 2013;24:2088–92. 10.1093/annonc/mdt140. 23592701 10.1093/annonc/mdt140

[12] Christensen TN, Andersen PK, Langer SW, Fischer BMB. Prognostic value of (18)F-FDG-PET parameters in patients with small cell lung cancer: a meta-analysis and review of current literature. Diagnostics. 2021. 10.3390/diagnostics11020174. 33530446 10.3390/diagnostics11020174 PMC7912276

[13] van Loon J, Offermann C, Ollers M, van Elmpt W, Vegt E, Rahmy A, et al. Early CT and FDG-metabolic tumour volume changes show a significant correlation with survival in stage I-III small cell lung cancer: a hypothesis generating study. Radiother Oncol. 2011;99:172–5. 10.1016/j.radonc.2011.03.014. 21571382 10.1016/j.radonc.2011.03.014 PMC4555839

[14] Christensen TN, Langer SW, Villumsen KE, Johannesen HH, Lofgren J, Keller SH. 18F-fluorothymidine (FLT)-PET and diffusion-weighted MRI for early response evaluation in patients with small cell lung cancer: a pilot study. Eur J Hybrid Imaging. 2020;4(1):2. 10.1186/s41824-019-0071-5. 34191195 10.1186/s41824-019-0071-5 PMC8218141

[15] Guberina M, Poettgen C, Metzenmacher M, Wiesweg M, Schuler M, Aigner C, et al. Prognostic value of Post-Induction chemotherapy volumetric Pet/Ct parameters for stage Iiia/B Non-Small cell lung cancer patients receiving definitive chemoradiotherapy. J Nucl Med. 2021;62:1684–91. 10.2967/jnumed.120.260646. 34016730 10.2967/jnumed.120.260646 PMC8612197

[16] Boellaard R, Delgado-Bolton R, Oyen WJ, Giammarile F, Tatsch K, Eschner W, et al. FDG PET/CT: EANM procedure guidelines for tumour imaging: version 2.0. Eur J Nucl Med Mol Imaging. 2015;42:328–54. 10.1007/s00259-014-2961-x. 25452219 10.1007/s00259-014-2961-x PMC4315529

[17] Vaz SC, Adam JA, Delgado Bolton RC, Vera P, van Elmpt W, Herrmann K, et al. Joint EANM/SNMMI/ESTRO practice recommendations for the use of 2-[(18)F]FDG PET/CT external beam radiation treatment planning in lung cancer V1.0. Eur J Nucl Med Mol Imaging. 2022;49:1386–406. 10.1007/s00259-021-05624-5. 35022844 10.1007/s00259-021-05624-5 PMC8921015

[18] Leitlinienprogramm Onkologie (Deutsche Krebsgesellschaft DK, AWMF). Prävention, Diagnostik, Therapie und Nachsorge des Lungenkarzinoms, Lang- version 4.0, 2025, AWMF-Registernummer:020-007OLhttps://www.leitlinienpro-gramm-onkologie.de/leitlinien/lungenkarzinom/; Zugriff am [25.07.2025]. 2025.

[19] Erdi YE, Mawlawi O, Larson SM, Imbriaco M, Yeung H, Finn R, et al. Segmentation of lung lesion volume by adaptive positron emission tomography image thresholding. Cancer. 1997;80:2505–9. 10.1002/(sici)1097-0142(19971215)80:12+%3C2505::aid-cncr24%3E3.3.co;2-b. 9406703 10.1002/(sici)1097-0142(19971215)80:12+<2505::aid-cncr24>3.3.co;2-b

[20] Huang YE, Chen CF, Huang YJ, Konda SD, Appelbaum DE. Interobserver variability among measurements of the maximum and mean standardized uptake values on (18)F-FDG PET/CT and measurements of tumor size on diagnostic CT in patients with pulmonary tumors. Acta Radiol. 2010;51:782–8. 10.3109/02841851.2010.497772. 20707663 10.3109/02841851.2010.497772

[21] van Gomez Lopez O, Garcia Vicente AM, Honguero Martinez AF, Soriano Castrejon AM, Jimenez Londono GA, Udias JM, et al. Heterogeneity in [(1)(8)F]fluorodeoxyglucose positron emission tomography/computed tomography of non-small cell lung carcinoma and its relationship to metabolic parameters and pathologic staging. Mol Imaging. 2014;13. 10.2310/7290.2014.00032. 10.2310/7290.2014.00032 25248853

[22] Schemper M, Smith TL. A note on quantifying follow-up in studies of failure time. Control Clin Trials. 1996;17:343–6. 10.1016/0197-2456(96)00075-x. 8889347 10.1016/0197-2456(96)00075-x

[23] Gronberg BH, Killingberg KT, Flotten O, Bjaanaes MM, Brustugun OT, Madebo T, et al. High-Dose versus Standard-Dose Twice-Daily thoracic radiotherapy in Limited-Stage SCLC: final survival Data, Long-Term Toxicity, and relapse patterns in a Randomized, Open-Label, phase II trial. J Thorac Oncol. 2025. 10.1016/j.jtho.2025.04.007. 40258573 10.1016/j.jtho.2025.04.007

[24] Herz A, Guberina M, Pottgen C, Gauler T, Ton TTM, Fischedick G, et al. The effect of durvalumab consolidation after definitive radiochemotherapy for non-operable stage III non-small cell lung cancer on the dose effect relation for therapy related pulmonary infiltrates as a risk factor for pneumonitis. Transl Lung Cancer Res. 2025;14:2074–88. 10.21037/tlcr-2024-1284. 40673077 10.21037/tlcr-2024-1284 PMC12261351

[25] Kim H, Yoo IR, Boo SH, Park HL, O JH, Kim SH. Prognostic value of pre- and post-treatment FDG PET/CT parameters in small cell lung cancer patients. Nucl Med Mol Imaging. 2018;52:31–8. 10.1007/s13139-017-0490-9. 29391910 10.1007/s13139-017-0490-9 PMC5777958

[26] Choi EK, Park M, Im JJ, Chung YA, Oh JK. Prognostic value of (18)F-FDG PET/CT metabolic parameters in small cell lung cancer. J Int Med Res. 2020;48:300060519892419. 10.1177/0300060519892419. 31880209 10.1177/0300060519892419 PMC7607737

[27] Chang H, Lee SJ, Lim J, Lee JS, Kim YJ, Lee WW. Prognostic significance of metabolic parameters measured by (18)F-FDG PET/CT in limited-stage small-cell lung carcinoma. J Cancer Res Clin Oncol. 2019;145:1361–7. 10.1007/s00432-019-02848-9. 30900157 10.1007/s00432-019-02848-9 PMC11810363

[28] Fu L, Zhu Y, Jing W, Guo D, Kong L, Yu J. Incorporation of circulating tumor cells and whole-body metabolic tumor volume of (18)F-FDG PET/CT improves prediction of outcome in IIIB stage small-cell lung cancer. Chin J Cancer Res. 2018;30:596–604. 10.21147/j.issn.1000-9604.2018.06.04. 30700928 10.21147/j.issn.1000-9604.2018.06.04 PMC6328501

[29] Pottgen C, Gauler T, Bellendorf A, Guberina M, Bockisch A, Schwenzer N. Standardized uptake decrease on [18F]-fluorodeoxyglucose positron emission tomography after neoadjuvant chemotherapy is a prognostic classifier for long-term outcome after multimodality treatment: secondary analysis of a randomized trial for resectable stage IIIA/B non–small-cell lung cancer. J Clin Oncol. 2016;34:2526–33. 10.1200/JCO.2015.65.5167. 27247220 10.1200/JCO.2015.65.5167

[30] Guberina M, Guberina N, Pottgen C, Gauler T, Richlitzki C, Metzenmacher M, et al. Effectiveness of durvalumab consolidation in stage III non-small-cell lung cancer: focus on treatment selection and prognostic factors. Immunotherapy. 2022;14:927–44. 10.2217/imt-2021-0341. 35822656 10.2217/imt-2021-0341

[31] Guberina M, Darwiche K, Hautzel H, Ploenes T, Pottgen C, Guberina N, et al. Impact of EBUS-TBNA in addition to [(18)F]FDG-PET/CT imaging on target volume definition for radiochemotherapy in stage III NSCLC. Eur J Nucl Med Mol Imaging. 2021;48:2894–903. 10.1007/s00259-021-05204-7. 33547554 10.1007/s00259-021-05204-7 PMC8263445

[32] Guberina M, Herrmann K, Pottgen C, Guberina N, Hautzel H, Gauler T, et al. Prediction of malignant lymph nodes in NSCLC by machine-learning classifiers using EBUS-TBNA and PET/CT. Sci Rep. 2022;12:17511. 10.1038/s41598-022-21637-y. 36266403 10.1038/s41598-022-21637-y PMC9584941

[33] Hautzel H, Alnajdawi Y, Fendler WP, Rischpler C, Darwiche K, Eberhardt WE, et al. N-staging in large cell neuroendocrine carcinoma of the lung: diagnostic value of [(18)F]FDG PET/CT compared to the histopathology reference standard. EJNMMI Res. 2021;11:68. 10.1186/s13550-021-00811-9. 34292419 10.1186/s13550-021-00811-9 PMC8298649

[34] Berghmans T, Lievens Y, Aapro M, Baird AM, Beishon M, Calabrese F, et al. European cancer organisation essential requirements for quality cancer care (ERQCC): lung cancer. Lung Cancer. 2020;150:221–39. 10.1016/j.lungcan.2020.08.017. 33227525 10.1016/j.lungcan.2020.08.017

